# Automated human-level diagnosis of dysgraphia using a consumer tablet

**DOI:** 10.1038/s41746-018-0049-x

**Published:** 2018-08-31

**Authors:** Thibault Asselborn, Thomas Gargot, Łukasz Kidziński, Wafa Johal, David Cohen, Caroline Jolly, Pierre Dillenbourg

**Affiliations:** 1 CHILI Lab, EPFL, Route Cantonale, 1015 Lausanne, Switzerland; 2 Psychiatrie de l’Enfant et de l’Adolescent, Pitié Salpêtriére - Charles Foix, Assistance Publique Hôpitaux de Paris, 47/83 boulevard de l’Hôpital, 75013 Paris, France; 3 ISIR, Sorbonne Université, 4 Place Jussieu, 75005 Paris, France; 4 CHART Laboratory - EA 4004, TIM, Paris 8 University, 93526 Saint Denis, France; 5 Department of Bioengineering, Stanford University, 443 Via Ortega, Stanford, CA 94305 USA; 6 LSRO Lab, EPFL, Route Cantonale, 1015 Lausanne, Switzerland; 7 LPNC, Univ. Grenoble Alpes, 38040 Grenoble, France; 8 CNRS, LPNC UMR 5105, 38040 Grenoble, France

**Keywords:** Diagnosis, Patient education

## Abstract

The academic and behavioral progress of children is associated with the timely development of reading and writing skills. Dysgraphia, characterized as a handwriting learning disability, is usually associated with dyslexia, developmental coordination disorder (dyspraxia), or attention deficit disorder, which are all neuro-developmental disorders. Dysgraphia can seriously impair children in their everyday life and require therapeutic care. Early detection of handwriting difficulties is, therefore, of great importance in pediatrics. Since the beginning of the 20th century, numerous handwriting scales have been developed to assess the quality of handwriting. However, these tests usually involve an expert investigating visually sentences written by a subject on paper, and, therefore, they are subjective, expensive, and scale poorly. Moreover, they ignore potentially important characteristics of motor control such as writing dynamics, pen pressure, or pen tilt. However, with the increasing availability of digital tablets, features to measure these ignored characteristics are now potentially available at scale and very low cost. In this work, we developed a diagnostic tool requiring only a commodity tablet. To this end, we modeled data of 298 children, including 56 with dysgraphia. Children performed the BHK test on a digital tablet covered with a sheet of paper. We extracted 53 handwriting features describing various aspects of handwriting, and used the Random Forest classifier to diagnose dysgraphia. Our method achieved 96.6% sensibility and 99.2% specificity. Given the intra-rater and inter-rater levels of agreement in the BHK test, our technique has comparable accuracy for experts and can be deployed directly as a diagnostics tool.

## Introduction

Despite a broad use of laptops and tablets in schools, handwriting remains an important skill to be acquired during childhood education as it is the basis of core educational activities, such as taking notes, composition, and self-expression.^1–3^ Handwriting is a complex task as it involves attentional, perceptual, linguistic, and fine motor skills.^4–6^ That is why, even in normally developing children, learning handwriting spans a period of 10 years, between the ages of 5 (preschool) and 15.^7,8^ During this time, handwriting evolves initially on a qualitative level (legibility) and then on a quantitative level (speed).^9–11^

Even with correct training, between 5 and 34% of children never master handwriting.^11,12^ With the rising cognitive demand of school work as they progress through school, these children quickly face more general difficulties. As they encounter trouble automatizing their handwriting, they cannot handle simultaneous tasks, such as grammar, spelling, and composition. This leads to an increase in fatigue and decreases in cognitive performance and self-esteem.^13–16^ Hence, it is of prime importance to detect and remediate any handwriting difficulties as early as possible.^5,17^

The most established and adopted classification of dysgraphia was proposed by Deuel.^18^ Deuel distinguishes three sub-types of this disorder: (1) dyslexic dysgraphia, which appears when spontaneously written text is illegible while the copy of a written text is relatively preserved; (2) spatial dysgraphia, which is due to a defect in the understanding of space and characterized by illegible writing, whether spontaneously produced or copied, while handwriting velocity remains normal; and (3) motor dysgraphia, which appears when both spontaneously written and copied text may be illegible, reflecting motor impairments. In this type of dysgraphia, the handwriting velocity as well as the drawing are abnormal.

Many quantitative tests have been proposed to evaluate penmanship. Most quantitative methods assess handwriting according to several predefined, specific criteria. Experts then grade these criteria and add up the sub-scores. A number of tests using this principle have been developed for different alphabets. Most of the tests which assess dysgraphia are based on a copying task (see Table 1), meaning that dyslexic dysgraphia cannot be detected using these tests.

**Table 1:** Overview of the different tests used to diagnose dysgraphia

	Validation number	Age range [years old]	Test duration [min]	Scoring duration [min]	Alphabet	Language	Number of items	Dynamic of handwriting	Pressure	Tilt	Speed	Posture	Writing task
Ajuriaguerra^28^	350	6–12	2	5	Latin	French	37	✗	✓^a^	✗	✓	✓	WT1
BHK^29^	837	6–12	5	10	Latin	Multi-language	13	✗	✗	✗	✓	✗	WT2
BHK-teenager^30^	471	12–18	5	10	Latin	Multi-language	9	✗	✗	✗	✓	✗	WT2
DASH^31^	546	9–16	20	10	Latin	English	5	✗	✗	✗	✓	✗	WT3
HHE^32^	230	6–18	5	0	Hebrew	Hebrew	10	✗	✗	✗	✗	✗	WT4

WT1: copy a sentence several times, request of quality and speed; WT2: copy a long text for 5 min; WT3: copy a sentence several times, alphabet, geometric figures, and composition; and WT4: copy a text containing all letters

Ajuriaguerra scale (E scale): is a well-spread test evaluating the quality of the writing depending on speed and precision. It has a special focus on the posture and style of pen grasp of the child

Concise Evaluation Scale for Children’s Handwriting (BHK): is the gold standard test with which to diagnose dysgraphia in a Latin-alphabet-based language^28–30^

BHK for teenagers: has also been created using the same principles

Detailed Assessment of Speed of Handwriting (DASH test): evaluates the quality and speed of writing under different conditions (quality, speed, writing about a free topic of the child’s choice)

Hebrew Handwriting Evaluation (HHE): examines Hebrew handwriting products and assesses the legibility through both global and analytic measures

^a^Some pressure aspects of handwriting are assessed thanks to carbon paper

In Table 1, we summarize the different tests used widely to diagnose dysgraphia. As shown, these tests are heterogeneous as they were designed specifically to assess the handwriting quality for a specific alphabet or age range. Moreover, we can see that these tests are based on handwriting from different writing tasks (see the core task column in Table 1), which might imply high variability in the results. Finally, an important part of the overall handwriting process is not taken into account. Only the final product of handwriting is used for analysis, disregarding the handwriting dynamic, tilt, and, in most cases, pressure.

One of the main drawbacks of these tests is that the scoring of several parameters relies on human judgment, which makes testing more subjective. Moreover, grading the BHK test is also time-consuming since scoring can take up to 15 min. Additionally, as the expert responsible for the scoring only has access to the final static image of the child’s handwriting, some very informative handwriting aspects, such as the handwriting dynamics, pressure between the pen and tablet, and pen tilt, remains hidden and are, therefore, not used in the diagnosis. In the same way, posture and grasping style are difficult to assess and must be done live by an expert evaluator. Finally, the text used in the test is standardized (the content of the text is always the same). Consequently, the test cannot be performed during ecological writings sessions (e.g., during school sessions with the text actually written everyday by the child).

The rapid development of digital tablets in the last decade allowed us to tackle partially some of these problems. It made possible the evaluation not only of the final product of handwriting (the static image), but also its dynamics. Multiple studies have employed these new technologies to better understand writing disabilities. Pagliarini et al.^19^ used tablets to collect data on handwriting ability before handwriting is performed automatically. Quantitative methods allowed them to find patterns indicating potential future writing impairments at a very early age. Mekyska et al.^20^ used a Random Forest model to classify dysgraphic children. The authors included 54 third-grade Israeli children in the study and used a 10-item questionnaire for Hebrew handwriting proficiency (HPSQ)^21^ to identify poor writing. In the adult population, automatic handwriting assessment tools were proposed for Parkinson’s Disease as a potential biomarker.^22^

In this work, we build on previous work in order to design a digital diagnostic tool. Compared to previously established results, we focus on clinical relevance in pediatrics. To this end, we analyzed data for children who have been clinically diagnosed with dysgraphia, and matched them with a cohort of children with typical development. We maximized the potential impact of the work by focusing on the Latin alphabet—the most popular script worldwide, which is used by approximately 2.6 billion people.

Moreover, we defined features related to those currently used in clinical practice. Our quantitative model leverages four categories of writing characteristics: the geometrical aspect of handwriting, and the use of pressure, tilt, and kinematics. We used a Random Forest classifier to predict dysgraphia. In the test set, approximately 96% of dysgraphic writers were labeled correctly (true positive ratio), while less than 1% of non-dysgraphic children were incorrectly diagnosed (false negative ratio). We obtained an F1-score of 97.98%.

After building the model, we explored and analyzed the most important features for the diagnosis of dysgraphia. In this analysis, we combined statistical analysis and collaboration with clinicians, exchanging examples and comments. The conclusions were then used to provide insights for the development of a new screening tool that would modernize the current gold-standard test, BHK.

## Results

As described previously, our database is not balanced in terms of positive and negative examples (242 *TD* children versus 56 *D* children), which can skew the model towards a larger subpopulation.

In order to validate the accuracy, we divided our data into two disjoint sets.

- *Training set* —70% of *TD dataset* and 70% of *D dataset*.
- *Testing set* —30% of *TD dataset* and 30% of *D dataset*.

A *k*-fold cross-validation^23^ (with *k* = 25) on a Random Forest classifier^24^ was performed in order to test our model.

Due to the differences between positive (dysgraphic children) and negative examples (non-dysgraphic children) in the database, reporting the overall accuracy might be misleading (a model that always predicts non-dysgraphia will be ~75% accurate). Following machine learning literature, we report the F1-Score. The F1-Score is the harmonic mean of Precision and Recall. Therefore, the score takes both false positives and false negatives into account, making it more comparable across studies with different proportions of classes. The F1-score is defined as

$$F1 - Score = 2 \cdot \frac{{Precision \cdot Recall}}{{Precision + Recall}}$$

where

$$\begin{array}{l}Recall = \frac{{True{\kern 1pt} Positive}}{{True{\kern 1pt} Positive + False{\kern 1pt} Negative}}\,{\mathrm{and}}\cr Precision = \frac{{True{\kern 1pt} Positive}}{{True{\kern 1pt} Positive + False{\kern 1pt} Positive}}.\end{array}$$

In our case, the *True Positive* ratio corresponds to the proportion of dysgraphic children correctly labeled dysgraphic, while the *False Negative* ratio refers to the proportion of dysgraphic children incorrectly labeled non-dysgraphic. Finally, the *False Positive* ratio defines the proportion of non-dysgraphic children incorrectly labeled dysgraphic.

For our model, after the 25-fold cross-validation, we obtained a F1-score of 97.98% (Std. of 2.68%). We found this result very satisfactory given the small number of dysgraphic recordings used for training the model. We also conjectured that a larger sample would improve the generalizability and robustness of the model.

### Robustness of the test

**Fig. 1:**
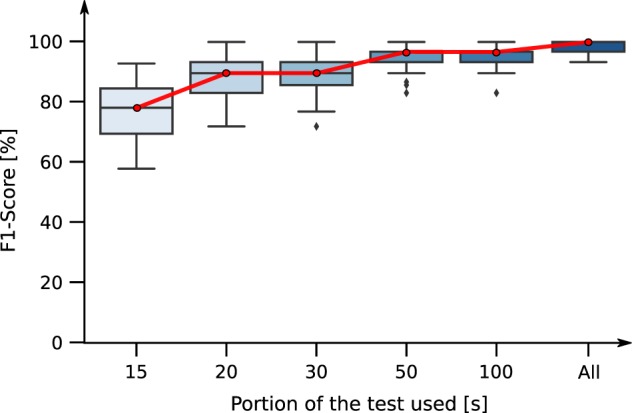
Box plot representing the F1-score as a function of time period of the test used for training. We used Random Forest for classification, and each model was trained following the same *k*-fold, cross-validation procedure with *k* = 25

To validate the robustness of the test, we measured how much data per user was needed in order to accurately predict dysgraphia. To that end, we trained the model, using only the first seconds of the test, while keeping the same workflow of training, including the *k*-fold validation (*k* = 25). In Fig. 1, we present the F1-score as a function of the length of the portion of the test used to train the model. For example, 15 s means that only the data recorded during the first 15 s of the BHK test were used to train the model. We can see that, after 15 s of testing, the results are already satisfactory (F1-Score of 77.21%), but the high standard deviation (10.34%) may indicate that the model is not generalizable. After 50 s of testing, the F1-Score reaches 93.93%, while the standard deviation drops to 4.6%. For any longer periods of time, the results improve only marginally.

We believe that this result indicates the robustness of our features. Indeed, even with the noise coming with the restricted portion (for example, 15 s) of the test used (a smaller number of examples means less statistical significance), our model still manages to extract relevant information from the features measured. This is an indirect benefit compared to the BHK test, which must be interpreted in its entirety.

### Discriminative features

In order to analyze the most discriminative features, we first sort them by importance. Following machine learning literature, we use one of the most popular choices, Gini importance.^24^ Table 2 presents the eight most important features (i.e., the most discriminative) emerging the Random Forest model (averaged over 25 folds). Features related to frequencies seem to be very discriminative as six out of eight of the most important features are related to frequencies. Features from all four of our categories are represented among the eight most discriminative features: three are kinematic features, three represent tilt, one is related to pen pressure, and one is a static feature. In the next section, we will analyze these features further.

**Table 2:** The most important features found by the Random Forest model, using Gini importance as a metric

**Rank**	**Category**	**Name**	**Importance (Std.) [%]**
1	Kinematic	Median of Power Spectral of Speed Frequencies	15.71 (9.06)
2	Kinematic	Bandwidth of Speed Frequencies	12.08 (8.00)
3	Pressure	Mean Speed of Pressure Change	9.81 (6.52)
4	Static	Space Between Words	7.45 (6.73)
5	Tilt	Distance to Mean of Speed of Tilt-X Change Frequencies	6.07 (4.30)
6	Kinematic	Distance to Mean of Speed Change Frequencies	5.18 (4.73)
7	Tilt	Bandwidth of Speed of Tilt-X Change Frequencies	4.10 (4.64)
8	Tilt	Median of Power Spectral of Tilt-Y Change Frequencies	2.97 (3.33)

We report the ranks, feature categories, and their importance averaged for the 25 folds and the standard deviation of importance over all folds

We notice that only one of these features, the space between words, could be extracted if we only had access to the final output of handwriting. This reassures us concerning the value of the digital tablet in assessing handwriting as it provides us access to important information previously inaccessible to clinicians analyzing standard tests, such as the BHK.

## Discussion

### Clinical features analysis

**Fig. 2:**
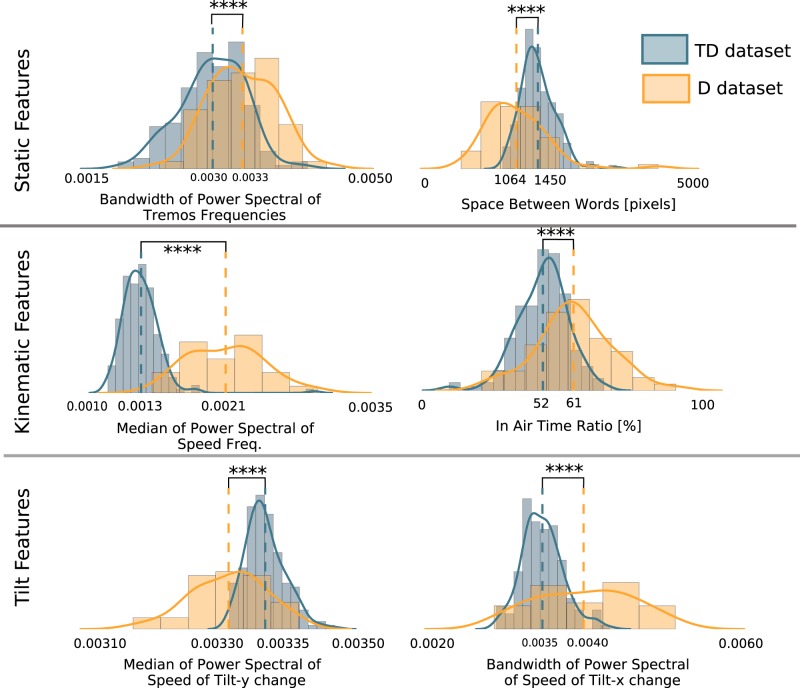
Distribution of the dysgraphic children (*D* dataset) and the non-dysgraphic children (*TD* dataset). For static features: *Bandwidth of Tremors Frequencies* and the *Space Between Words* features. For kinematic features: *Median of Power Spectral of Speed Frequencies* and the *In Air Time Ratio* features. For tilt features: *Median of Power Spectral of Speed of Tilt-y change* and the *Bandwidth of Power Spectral of Speed of Tilt-x change* features

**Fig. 3:**
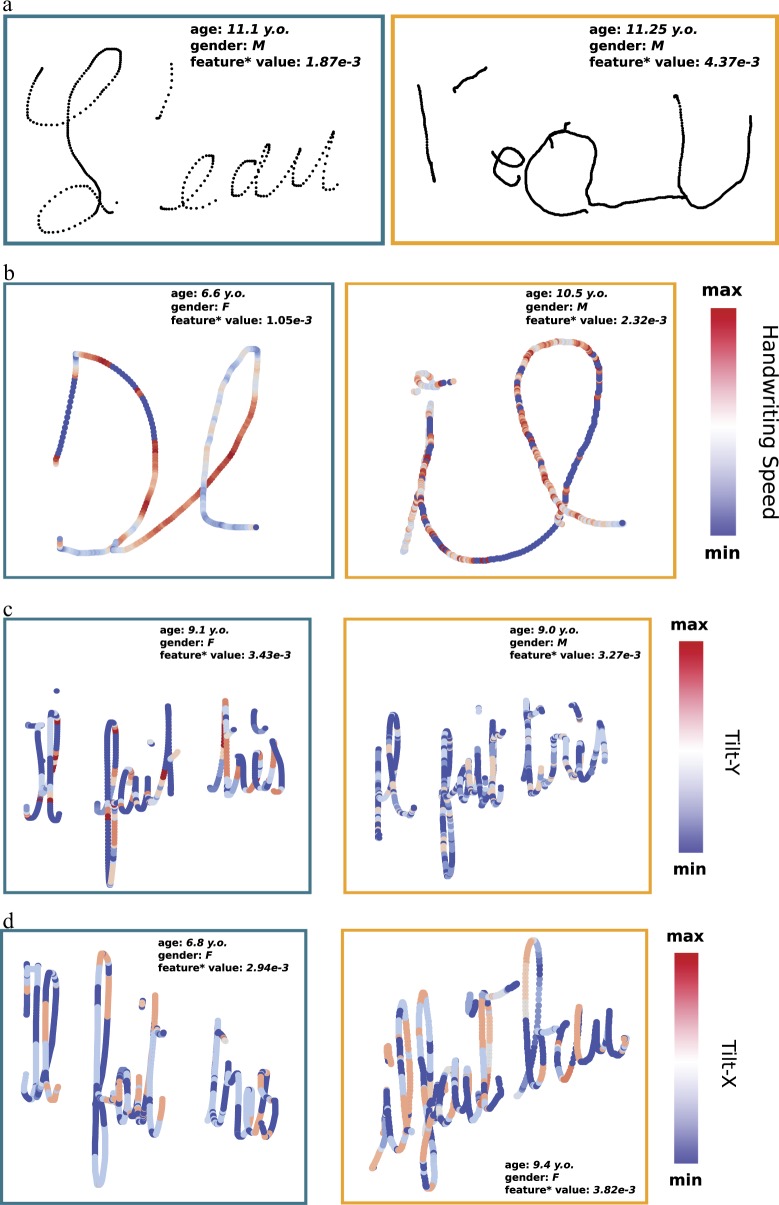
A comparison of different metrics for a non-dysgraphic child (left) and a child with dysgraphia (right)

The most discriminative static feature we found was the *Bandwidth of Tremor Frequencies* (see left graph of Fig. 2). This feature represents the range of tremor frequencies found in the handwriting of the writer under investigation. A high value for this feature means that many tremors were extracted from the handwriting. In Fig. 3a, we present an example of handwriting from a non-dysgraphic child (on the left) and a dysgraphic child (on the right). The handwriting of the non-dysgraphic child appears to be smooth. Conversely, the handwriting of the dysgraphic writer is not smooth; we can see easily some high-frequency shaking (as in the apostrophe or at the end of the “*a*”). We hypothesize that this characteristic results in an important value of the *Bandwidth of Tremors Frequencies* feature. The dysgraphic child hesitates more when forming letters, and it is harder for them to control the pen smoothly. This lack of smoothness is indicative of poor motor control, resulting in more noise. Interestingly, this feature is related to the BHK item *hesitation and shaking*. According to the therapists, a score of 0 (highest score) was obtained by the non-dysgraphic child for this feature, while a score of 3/5 (a low score) was obtained by the dysgraphic child.

Concerning the *Space Between Words* feature, we can see in the right graph of Fig. 2 that the non-dysgraphic tends to put more space between the words they write. This is related to the BHK item called *narrow words*. This BHK item indicates pathology if it is not possible to insert the letter “*o*” between each pair of words, meaning that not enough space is left. Moreover, in the case of dysgraphia, the writing is barely automatized, leading to irregular spaces between words. This irregularity is attested to by the difference in standard deviation that we can observe between the two groups (for more details, see *Std. TD* and *Std. D* in Supplementary Table 2).

The most discriminative kinematic feature we found was the *Median of the Power Spectral of Speed Frequencies*. This feature indicates that the speed frequencies of dysgraphic children are shifted toward high frequencies (see left graph of Fig. 2). In Fig. 3b, we present an example of the handwriting of a non-dysgraphic child on the left and a dysgraphic child on the right. The color corresponds to the handwriting speed at the time the points were recorded. In the dysgraphic child’s handwriting, we observe very rapid changes in speed (rapid acceleration and deceleration), contrary to what can be found in the handwriting of the non-dysgraphic writer. It is interesting to note that the features linked with acceleration were not found to be discriminative as these sudden changes of speed are local (compensated for by long periods of constant speed). These sudden changes of speed are translated into high frequencies during the Fourier transformation. This feature relates the fact that we can find more saccades during the handwriting of the dysgraphic child due to the lack of automation and control in his/her hand movements. In the case of the BHK test, the only feature related to the kinematics of handwriting is the number of characters written after 5 min. The results of this very basic feature show that the dysgraphic children are slower than the non-dysgraphic ones.

Another interesting feature that was found to be discriminative was the *in-air time* (the proportion of time spent with the pen not touching the surface of the tablet), as can be seen in the right graph of Fig. 2. This result appears to be in line with previous findings.^25^

As can be seen in Fig. 2, the frequencies extracted from the speed of tilt change are very discriminative of dysgraphia. Concerning the Tilt-y, in contrast to what was observed for other categories of features, we can see that the non-dysgraphic children seem to exhibit higher frequencies during their handwriting. This finding is highlighted in Fig. 3c. For every point recorded, the color of the trace represents the speed of the tilt-y change. We can see that the dysgraphic child stays very constant, maintaining the tilt-y of his/her pen (almost no variations in the speed of the tilt-y change, with small absolute value). The non-dysgraphic child, in contrast, presents very rapid variations in his/her tilt-y change speed (rapid acceleration and deceleration). These very sudden variations are translated into high frequencies in the Fourier domain, shifting the median of the power spectral to high frequencies. Thus, we can infer that the non-dysgraphic child is able to change very frequently and quickly the tilt of his/her pen in the *y*-axis, whereas the dysgraphic child displays less tilt-y variation abilities, probably due to a more constraining and rigid pen grip.

Concerning the tilt-x, although the distribution of dysgraphic children is very spread out (see the right graph of Fig. 2), the dysgraphic children seem to present a larger range of frequencies in their handwriting concerning the speed of tilt-x change than seen typically in developing children. This means that they are not constant in the way they move their pen in the ZX plane (see Supplementary Figure 5 for more details). This finding is highlighted in Fig. 3d. For every point recorded, the color of the trace represents the speed of the tilt-x change. We can see more variations in the speed of tilt-x change for the dysgraphic child compared to that of the non-dysgraphic child, who seems to present more control in his/her movement. Contrary to the pen tilt in the direction perpendicular to the handwriting global direction (perpendicular to the lines of the paper sheet), proficient writers exhibit less variations (more control) in the speed of tilt change (and also the tilt, itself) in the direction of the lines of the paper sheet.

### Correlation between features

We analyzed correlations between pairs of 53 features extracted throughout this study. We found a strong, positive correlation between the *median of the power spectral of speed frequencies* and the *bandwidth of speed frequencies* (Pearson’s test: *r* = 0.96, *p* < 0.001), as well as between the median and the *distance to mean speed change frequencies* (Pearson’s test: *r* = 0.65, *p* < 0.001). In other words, these three features describe the same “abnormal” high-speed frequencies in the handwriting: the more a child’s frequencies differ from the average, the more these frequencies will be shifted towards high frequencies.

This finding encouraged us to test a simpler model, using only the *median of the power spectral of speed frequencies*. Despite the high correlation between the median and the bandwidth, adding the additional feature still has additional predictive power, as indicated by our cross-validation process. In particular, the model with both features presents a F1 score of 0.98 (Std. = 0.03), while the simplified model has an F1 score of 0.95 (Std. = 0.05). We conjecture that two correlated features, despite being linearly correlated, are still discriminative in a non-linear manner. Thanks to the robustness of the Random Forest in terms of correlations and non-linear structures, we obtained better results. We decided to report on the more accurate model, leaving the decision to decrease model complexity to the user.

### Main findings

We designed a method allowing clinical assessment of dysgraphia using a consumer tablet. Compared to existing tools, our method is cheaper, faster, free of human bias, validated on clinical data, and is applicable to Latin alphabet. The method leverages information contained not only in static writing but also in its dynamics. These characteristics make it useful not only as a clinical diagnostic tool, but also as a tool for parents or guardians to obtain high-quality assessment more frequently throughout development of the child. Granularity of the features allows to obtain more specific diagnosis and can lead to design of new exercises tailored to specific motor-impairments. In this section, we discuss accuracy, clinical relevance, applicability, and potential impact of our work.

On average, 96.6% (standard deviation of 5.02%) of the writers with dysgraphia were diagnosed correctly, while we achieved a 0.78% (standard deviation of 1.82%) false positive rate. The final model reached an F1-Score of 97.98% (standard deviation of 2.68%) Note that the inter-rater correlation in BHK is 0.89. Since our algorithm outperforms this value, we conclude that the algorithm learned to mimic the rater. These findings suggest that adding data from other raters should not only reduce bias, but also allow us to surpass the accuracy of each individual rater.

Our diagnostic system has the advantage of being almost costless (not including the cost of the tablet) and very fast (only a few milliseconds to deliver the diagnosis compared to 10 min for the BHK test). It also reduces subjectivity as the model is permeable to all the external parameters that can bias a human. Moreover, it is interesting to see that, among the 53 features used by our model, most of them are very technical and “low level”, i.e., measuring the mechanics of writing. In that way, our test is more robust to differences in handwriting style, language, and understanding of the text by the subject. Indeed, these features (for example, the three most discriminative features: the *Median of Power Spectral of speed frequencies*, the *Bandwidth of Speed Frequencies* and the *Mean Speed of Pressure Change*) can be interpreted the same way independently of the language or handwriting direction. For example, languages written from right to left, such as Hebrew, or from left to write, such as French, still share the same low-level characteristics. In future work, we envision testing the method for its robustness with other tests and, especially, other languages. Whenever the retraining of the model is needed, we are interested in validating it if an overlap between the most predictive features is large.

As the model includes 53 criteria of a child’s handwriting, the system helps us to build a more precise profile of the child compared to standard tests, in which only a few different criteria are available. Moreover, our model has the consequent advantage of not being restricted to the use of static features, such as in the current standard tests, but also uses kinematics, pressure, and tilt features. In the BHK test, the 13 items reflect *what is wrong* in the final product of the child’s handwriting, but do not give indications on *why it is wrong*. We believe that our system explores the handwriting pathology at a deeper level, and it permits analyzing the handwriting characteristics that lead to the imperfections seen in the final product. This brings with it potential therapeutic value, especially for remediation. Given new features, it is now possible to reach a more specific diagnosis rather than the general binary indication of dysgraphia. This will help clinicians focus on specific remediation exercises (e.g., exercises to increase the stability of the pen tilt or the change in pressure necessary for handwriting automation).

We demonstrated that we were able to use handwriting’s static, dynamic, pressure, and tilt features extracted from a digital tablet to diagnose dysgraphia very accurately. We believe that the knowledge gained from the analysis of the features extracted during this study can be applied to designing a new test to diagnose dysgraphia. This modernized test would have the consequent advantage of being able to assess the dynamic of handwriting, and the pressure of the pen as well as its tilt. Moreover, it would be possible to design this test with words maximizing the feature differences between dysgraphic and non-dysgraphic children.

## Method

### Participants

The present study was conducted in accordance with the Helsinki Declaration. It was approved by the Grenoble University Ethics Committee (Agreement No. 2016-01-05-79). It was conducted with the understanding and written consent of each child’s parents, the oral consent of each child, and in accordance with the ethics convention between the academic organization (Laboratoire de Psychologie et NeuroCognition (LPNC)—Centre National Recherche Scientifique) and educational organizations.

A total of 242 Typically Developing (TD) children were recruited in 14 primary schools from various Grenoble suburbs to ensure differing socio-economic environments (*TD dataset*). Children from the first to fifth grade were recruited from 43 classes. None of the TD children included in the study presented known learning disabilities or neuro-motor disorders.

The study also included 56 dysgraphic children (*D dataset*) recruited at the Learning Disorders Clinic of Grenoble Hospital (Centre Referent des Troubles du Langage et des Apprentissages, Centre-Hospitalier—Universitaire Grenoble). They were all diagnosed as dysgraphic based on their BHK scores. The scores were assigned by a single rater.

In order to validate the analysis on the combined dataset of *D* and *TD*, we needed to compare age distributions in both groups. The Kolmogorov–Smirnov test showed no statistical difference (*p* = 0.32) in terms of ages between the two distributions (*D* and *TD* datasets). Based on this result and the qualitative assessment of the Q–Q plot (see Supplementary Figure 6), we concluded that there was no evidence of a difference between these two distributions and they could be treated jointly in the analysis.

For more information concerning the participants, we refer the reader to Supplementary Table 1.

### Data collection

The 298 children (*TD dataset* + *D dataset*) involved in this study performed the BHK test by writing on a sheet of paper affixed to a Wacom graphic tablet (sampling frequency = 200 Hz; spatial resolution = 0.25 mm). A Wacom Intuos 4 tablet was used for the TD set, and a Wacom Intuos 3 for the D set. Pressure data were carefully calibrated between the two tablets.

The BHK test consists of copying a text for 5 min. The first five sentences of the text are composed of monosyllabic words typically learned during first grade. Then the complexity of the words starts increasing. Scoring includes two dimensions: (1) handwriting velocity, calculated by counting the number of characters written; and (2) handwriting fluency, which takes into account only the first five first sentences of the text and is assessed semi-quantitatively according to 13 clinical features (see Supplementary Table 6 for details on the features used).

The data was collected using Ductus software (LPNC laboratory).^26^ Doing so allowed children’s handwriting parameters to be saved, including the *x* and *y* coordinates, pressure, and tilt of the pen, for every time frame at a maximum sampling rate of 200 Hz. In addition, the age, gender, and laterality of the writers were saved.

The BHK tests of the dysgraphic children (*D dataset*) were rated by one expert from the hospital in Grenoble. None of the BHK tests from the *TD dataset* were rated for dysgraphia, which means that some of these children might be dysgraphic, as well.

### Features extraction

In this work, we tried to extract the spectrum of features that could describe handwriting in terms of different aspects, such as static, dynamic, tilt, and tremors.

We organized all the features into four categories:

- *Static features*—purely geometric characteristics of a written text.
- *Kinematic features*—dynamics of handwriting path.
- *Pressure features*—characteristics of the pressure recorded between the pen tip and the tablet surface.
- *Tilt features*—characteristics of the pen tilt.

Every feature used in the analysis is described below.

#### Static features

The design of the BHK and its scoring limit analysis to just the static aspects of handwriting. Each of the 13 BHK features can be classified into one of two categories. The first category regroups features which assess handwriting quality at a letter level. A direct translation of these features requires knowledge of the letter’s shape. Since this would require a large-scale analysis of shapes of letters, which would be language-dependent due to variations in the Latin alphabet, we disregarded these features in the analysis. The second category of features focuses on higher-level aspects of handwriting. In this case, we were able to construct features related to BHK concepts.

In this section, we outline the features engineered for the study. More details on the mapping between the BHK items and our static features can be found in Supplementary Table 6.

##### Space between words

The distance (in pixels) between words, averaged for the entire text and logged.

##### Handwriting density

A grid with 300-pixel cells covering the entire range of the handwriting trace was created. The number of points in each cell, if present, were stored in an array. The mean value of this array represented our approximation of the handwriting density.

##### Moment of handwriting

To compute this feature, we extracted bins of 300 points (from the same line of text) and computed its barycenter. The distance in the *y* direction between consecutive barycenters is computed and averaged for all of the points. This reflects the average direction of the written line, which could be a proxy for the “non-straight lines” item on the BHK.

##### Handwriting size

To compute this feature, we extracted bins of 300 points (from the same line of text) and computed the total surface occupied by the box bounding the trace.

##### Tremor frequencies

**Fig. 4:**
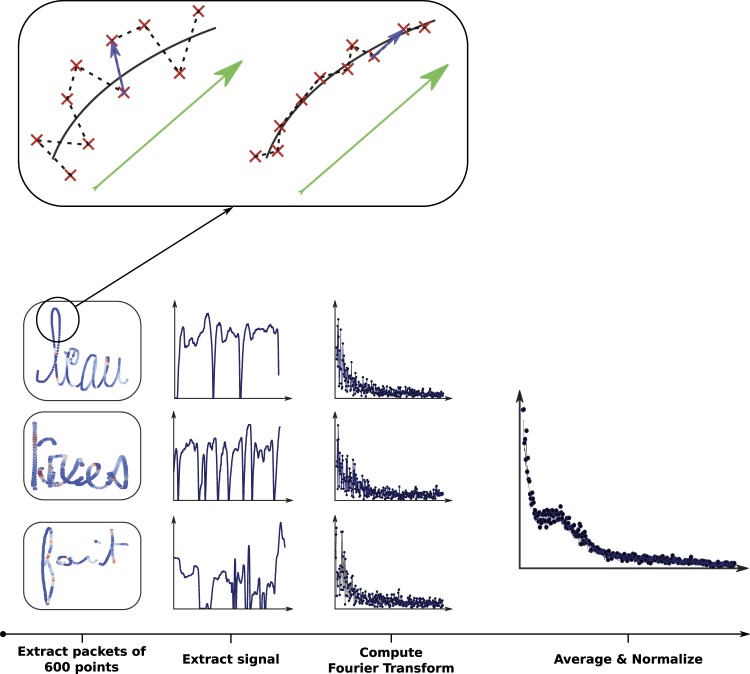
The whole process used to extract the frequency spectrum of our signal. (1) We first divided the BHK text into bins of 600 points. (2) For each packet, the signal was extracted. (3) We then computed the Fourier transform of the signal. (4) We took the average of all signals and finally performed a normalization. At the top of the figure is presented an example of a signal extracted from the data: the red dots are the point coordinates recorded by the device during handwriting. The vectors in blue are “local” vectors linking two consecutive points. The vector in green is the “global” vector (average of the nine blue vectors) representing the global direction of the handwriting. The cross product of these two vectors gives us an indication of the smoothness/shakiness of the handwriting. The image on the right comes from a writer with smoother/less shaky handwriting than the one on the left. The cross product operation will detect this difference

This feature quantifies shaky handwriting. For each child, we first divided the signal into bins of 600 points (as can be seen in Fig. 4) and extracted from each of these bins the deviance from the handwriting path. To do so, two types of vectors were extracted, as we present at the top of Fig. 4: for the first one, we computed a “global” vector by averaging bins of 10 points (represented in green in Fig. 4). This vector represents the global direction of the handwriting movement in a restricted area of 10 points. The second vector is local as it is not averaged on bins of points. It simply links points inside this restricted area of 10 points (represented in blue in Fig. 4). The cross product of these two vectors tells us how orthogonal the local vector is compared to the “global” vector. The greater the result of this operation is, the higher the deviance from the path is. We conjecture that shaky handwriting will result in local vectors being rarely aligned with their global counterparts and can then be detected with this method. For each of the 600 points, we log the norm of the cross product.

We computed the Fourier transform on the vectors, regrouping the results of all of these cross products. Then, the average of all of the Fourier transforms coming from these different bins of 600 points (see Fig. 4) was computed. In this manner, a normalization was finally achieved for every child in our database.

With this analysis, we aimed to quantify the tremor/shaky aspect of handwriting, which would then be translated by higher frequencies or a wider bandwidth in the spectral domain.

For example, we extracted the range of frequencies covering 90% of the spectral density. Our hypothesis is that, the smaller this value is (meaning that the distribution is more clustered), the more proficient the writer is. A writer having a huge bandwidth will not be fluent as they are less consistent in their movements. This feature is called *Bandwidth of Tremor Frequencies*.

Motivated by this concept, we also extract the median of the power spectral density. A higher value of this feature indicates a higher presence of high frequencies. We refer to this feature as *Median of Power Spectral of Tremor Frequencies*.

The last feature we define in this context is the distance between the spectral distribution of the writer to the averaged spectral distribution of all the writers in our database. The higher this distance is, the more eclectic the handwriting of this particular writer. This feature is called *Distance to Mean of Tremor Frequencies*.

#### Kinematic features

Detailed analysis of all kinematic features can be found in Supplementary Table 3.

##### Handwriting speed

We hypothesize that abnormal variability in speed is indicative of handwriting problems. We quantify the speed as the distance traveled by the pen divided by the time taken. Although Wacom data is collected at 200 Hz, we noticed high frequency noise, and, to remedy this issue, we applied a moving average filter with *n* = 10 and then subsampled every 10th point. We only kept the measurement if the pen stayed on the surface during the 10 points (no in-air time). Finally, we computed the *mean*, *maximum*, and *standard deviation* for each user.

With this technique, we had access to the local handwriting speed every 10 points. We then performed a linear regression to compute the evolution of the handwriting speed. Motivated by insights from clinicians, we also computed the *number of speed peaks per seconds*. To that end, we applied a Gaussian filter to the signal of velocity over time, and we computed the number of local maxima and minima extracted. We expect that the number of peaks should grow with the total duration of the test, and, therefore, we normalize this number by time.

##### Handwriting speed frequencies

We can interpret handwriting as a two-dimensional time series. As such, we can apply common time-series analysis techniques, and, in particular, we compute the Fourier transform. We conducted the process described in Fig. 4 and then we extracted the *Bandwidth of speed frequencies*, the *Median of power spectral of speed frequencies*, and the *Distance to mean of speed frequencies*.

##### Handwriting acceleration

Acceleration is another measure of variability in speed. We computed the *mean*, *maximum*, and *standard deviation* of acceleration following the same procedure as that used to extract the mean, maximum, and standard deviation of handwriting speed.

##### In-air time ratio

The in-air time ratio represents the proportion of time spent by the writer without touching the surface of the tablet. It was found to be a discriminative feature in a recent study interested in the analysis of dysgraphia.^25,27^

#### Pressure features

Detailed analysis of all pressure features can be found in Supplementary Table 4.

##### Pressure

The first features concerning the pressure are simply the *mean*, *maximum*, and *standard deviation* of the pressure.

##### Speed of pressure change

To compute the speed of pressure change, we used the same method we used for the speed of handwriting. We worked with averaged buckets of 10 points and divided the time spent by the difference between these two averaged bins of points. The *mean*, *maximum*, and *standard deviation* of these measures can then once again be extracted. The *number of peaks of speed of pressure change* during handwriting was also extracted. A Gaussian filter was applied to the signal and local minima and maxima of this filtered signal were extracted and normalized by the total amount of handwriting time (excluding the in-air time).

##### Speed of pressure change frequencies

The speed of pressure change can be seen as a time-series, and frequencies can be extracted using a Fourier transform. The same process as that described in Fig. 4 is followed to extract the *Bandwidth of speed of pressure change frequencies*, the *Median of power spectral of speed of pressure change frequencies*, and the *Distance to mean of speed of pressure change frequencies*.

#### Tilt features

Detailed analysis of all pressure features can be found in Supplementary Table 5.

The Wacom system logged the data measuring the pen tilt with two different angles, which we referred to in this paper as the Tilt-x and Tilt-y angles (see Supplementary Figure 5 for more details). Both angles are measured in the range between −60° and 60°. The tilt-x reflects the inclination of the pen in the direction of the written line, and the tilt-y reflects the inclination of the pen below the written line.

##### Tilt

Simple features were extracted for both angles, namely the *mean*, *maximum*, and *standard deviation* of the measurement.

##### Speed of tilt change

We computed the speed of tilt-x/tilt-y change in the same way as before, and we extracted the *mean*, *maximum*, *standard deviation*, and *number of peaks*. Finally, we also computed the evolution of the speed of tilt-x/tilt-y change over time.

##### Frequency of speed of tilt change

Using the same method as before, we computed the *Bandwidth of speed of tilt change frequencies*, the *Median of power spectral of speed of tilt change frequencies*, and the *Distance to mean of speed of tilt change frequencies*.

##### Code availability

Code is available from the corresponding author upon reasonable request.

## Electronic supplementary material

Supplemental files

## Data Availability

As it is possible to identify participants from the data, ethical requirements do not permit us to share participant data from this study.

## Electronic supplementary material

**Supplementary information** accompanies the paper on the *npj Digital Medicine* website (10.1038/s41746-018-0049-x).
