# Supplementary material for: Automated human-level diagnosis of dysgraphia using a consumer tablet
Source: NPJ Digit Med. 2018 Aug 31;1:42. doi: 10.1038/s41746-018-0049-x (PMC6550155; doi:10.1038/s41746-018-0049-x)
Supplement: Supplementary file 1 — Supplemental files [file 41746_2018_49_MOESM1_ESM.pdf]

Supplementary information

	Dataset	Grade 1	Grade 2	Grade 3	Grade 4	Grade 5
**Age (Std.) in years**	**TD**	6.77 (0.29)	7.80 (0.30)	8.75 (0.27)	9.85 (0.35)	10.90 (0.38)
	**D**	6.83 (0.10)	7.92 (0.67)	8.97 (0.53)	10.08 (0.67)	10.98 (0.61)
**Males/Females**	**TD**	23/26	22/29	21/20	22/26	28/25
	**D**	2/0	14/6	9/3	14/1	7/0
**Right handed/Left handed**	**TD**	41/8	48/3	38/3	41/7	46/7
	**D**	2/0	16/4	11/1	13/2	7/0

Supplementary Table 1: Summary statistics of the participants involved in this study.

	Feature	t-stat	p-value	Mean *TD*	Std. *TD*	Mean *D*	Std. *D*
**Static**	Moment of Handwriting	2.02	**0.045**	-25.02	73.45	-0.93	89.14
	Handwriting Density	2.10	**0.037**	84.48	42.40	99.31	65.36
	Space Between Words	-5.62	$\mathbf{5.36 \cdot 10^{-8}}$	0.0049	0.0016	0.0031	0.0029
	Handwriting Size	-2.89	**0.0042**	3686.53	1213.11	3145.91	1222.67
	Bandwidth of Tremor Freq.	5.92	$\mathbf{1.14 \cdot 10^{-8}}$	$3.00 \cdot 10^{-3}$	$3.92 \cdot 10^{-4}$	$3.36 \cdot 10^{-3}$	$3.86 \cdot 10^{-4}$
	Median of Power spectral of Tremor Freq.	5.81	$\mathbf{2.02 \cdot 10^{-8}}$	$3.18 \cdot 10^{-3}$	$1.77 \cdot 10^{-4}$	$3.32 \cdot 10^{-3}$	$1.03 \cdot 10^{-4}$
	Dist to mean of Tremor Freq.	3.65	$\mathbf{3.21 \cdot 10^{-4}}$	$2.57 \cdot 10^{-4}$	$1.52 \cdot 10^{-4}$	$3.56 \cdot 10^{-4}$	$2.38 \cdot 10^{-4}$

Supplementary Table 2: Statistics of static handwriting features in two groups of writers (*D dataset* and *TD dataset*). For each feature, we report the mean and standard deviation in each group as well as the t-statistic and p-value from the comparison between the two groups.

	Feature	t-stat	p-value	Mean *TD*	Std. *TD*	Mean *D*	Std. *D*
**Kinematic**	Mean Speed	-2.23	**0.027**	3.22	1.17	2.81	1.25
	Max Speed	2.81	**0.0054**	20.51	10.22	25.02	11.01
	Std. of Speed	2.17	**0.031**	2.50	0.78	2.78	1.07
	Increase (slope) of Speed	2.54	**0.012**	$1.65 \cdot 10^{-4}$	$3.37 \cdot 10^{-4}$	$3.41 \cdot 10^{-4}$	$6.98 \cdot 10^{-4}$
	Nb of Speed Peaks per secs	-0.70	0.49	0.038	$3.76 \cdot 10^{-3}$	0.037	$2.20 \cdot 10^{-3}$
	Mean Acceleration	-1.37	0.17	0.40	0.17	0.36	0.17
	Max Acceleration	-0.72	0.47	6.17	16.34	4.58	2.12
	Std. of Acceleration	-0.22	0.83	0.43	0.30	0.42	0.17
	Increase of Acceleration	-0.40	0.69	$4.67 \cdot 10^{-5}$	$2.35 \cdot 10^{-3}$	$-8.51 \cdot 10^{-5}$	$1.10 \cdot 10^{-3}$
	Bandwidth of Speed Freq.	20.86	$\mathbf{4.92 \cdot 10^{-55}}$	$1.20 \cdot 10^{-3}$	$1.97 \cdot 10^{-4}$	$2.19 \cdot 10^{-3}$	$5.24 \cdot 10^{-4}$
	Median of Power Spectral of Speed Freq.	21.49	$\mathbf{5.12 \cdot 10^{-57}}$	0.0013	$1.83 \cdot 10^{-4}$	0.0021	$3.34 \cdot 10^{-3}$
	Dist to mean of Speed Freq.	8.60	$\mathbf{1.23 \cdot 10^{-15}}$	$2.84 \cdot 10^{-4}$	$1.39 \cdot 10^{-4}$	$5.62 \cdot 10^{-4}$	$3.49 \cdot 10^{-4}$
	In Air Time Ratio	6.94	$\mathbf{3.91 \cdot 10^{-11}}$	0.52	0.075	0.61	0.11

Supplementary Table 3: A comparison between the two groups of writers (*D dataset* and *TD dataset*) for every feature based on the handwriting kinematics. For each feature, we report the mean and standard deviation of the two groups as well as the associated t-statistic and p-value for the t-test.

	**Feature**	**t-stat**	**p-value**	**Mean *TD***	**Std. *TD***	**Mean *D***	**Std. *D***
**Pressure**	Mean Pressure	1.10	0.27	493.25	116.79	676.24	2193.71
	Max Pressure	1.56	0.12	968.27	93.26	1386.66	3540.76
	Std. of Pressure	1.53	0.13	203.84	39.54	283.80	686.99
	Mean Speed of Pressure Change	-9.01	$\mathbf{7.50 \cdot 10^{-17}}$	0.23	0.091	0.015	0.26
	Max Speed of Pressure Change	-2.96	**0.0034**	13.83	3.77	10.69	12.35
	Std. of Speed of Pressure Change	-0.29	0.77	2.50	0.67	2.37	5.79
	Increase of Speed of Pressure Change	2.71	**0.0070**	$-2.41 \cdot 10^{-5}$	$4.89 \cdot 10^{-5}$	$-4.32 \cdot 10^{-6}$	$4.21 \cdot 10^{-5}$
	Nb of Peaks of Speed of Pressure Change	-8.39	$\mathbf{4.63 \cdot 10^{-15}}$	0.0035	$6.9 \cdot 10^{-4}$	0.0026	$6.72 \cdot 10^{-4}$
	Bandwidth of Speed of Pressure Change Freq.	-0.46	0.65	0.0017	$1.93 \cdot 10^{-4}$	0.0017	$2.29 \cdot 10^{-4}$
	Median of Power Spectral of Speed of Pressure Change Freq.	3.20	**0.0016**	0.0022	$1.60 \cdot 10^{-4}$	0.0023	$2.16 \cdot 10^{-4}$
	Dist to Mean of Speed of Pressure Change Freq.	4.87	$\mathbf{2.09 \cdot 10^{-6}}$	$1.72 \cdot 10^{-4}$	$6.26 \cdot 10^{-5}$	$2.31 \cdot 10^{-4}$	$1.15 \cdot 10^{-4}$

Supplementary Table 4: A comparison between the two groups of writers (*D dataset* and *TD dataset*) for every feature based on the pressure between the pen and tablet surface. For each feature, we compute the mean and standard deviation of the two groups as well as the t-statistic and p-value for the t-test.

	Feature	F statistic	p-value	Mean *TD*	Std. *TD*	Mean *D*	Std. *D*
**Tilt**	Mean Tilt-x	-1.67	0.096	607.10	53.01	592.90	61.48
	Mean Tilt-y	-0.29	0.77	1233.29	629.65	1204.48	669.94
	Max Tilt-x	2.72	**0.0071**	727.74	75.17	759.64	79.62
	Max Tilt-y	5.73	**$3.02 \cdot 10^{-8}$**	1788.75	819.03	2517.14	843.35
	Std. of Tilt-x	3.56	**$4.55 \cdot 10^{-4}$**	40.43	14.25	49.00	19.40
	Std. of Tilt-y	0.64	0.52	169.59	269.26	193.70	131.81
	Mean Speed of Tilt-x change	-1.60	0.11	0.024	0.014	0.021	0.012
	Mean Speed of Tilt-y change	-1.15	0.25	0.031	0.15	0.0077	0.043
	Max Speed of Tilt-x change	-1.10	0.27	1.66	0.76	1.54	0.68
	Max Speed of Tilt-x change	1.41	0.16	8.38	18.89	12.74	23.43
	Std. of Speed of Tilt-x change	3.18	**0.0017**	0.18	0.04	0.20	0.037
	Std. of Speed of Tilt-y change	-0.61	0.54	1.04	2.55	0.82	1.16
	Increase of Speed of Tilt-x change	0.29	0.77	$6.32 \cdot 10^{-7}$	$6.16 \cdot 10^{-6}$	$9.17 \cdot 10^{-7}$	$7.03 \cdot 10^{-6}$
	Increase of Speed of Tilt-y change	-0.98	0.33	$9.89 \cdot 10^{-6}$	$9.66 \cdot 10^{-5}$	$-2.83 \cdot 10^{-6}$	$1.36 \cdot 10^{-5}$
	Nb of peaks of Tilt-x Speed	-0.47	0.64	1174.56	235.35	1156.01	307.94
	Nb of peaks of Tilt-y Speed	-1.30	0.19	1093.54	220.77	1045.73	285.59
	Bandwidth of Speed of Tilt-x Change Freq.	9.08	**$4.73 \cdot 10^{-17}$**	$3.42 \cdot 10^{-3}$	$2.42 \cdot 10^{-4}$	$3.92 \cdot 10^{-3}$	$5.74 \cdot 10^{-4}$
	Median of Power Spectral of Speed of Tilt-x Change Freq.	1.33	0.19	$3.34 \cdot 10^{-3}$	$2.49 \cdot 10^{-5}$	$3.35 \cdot 10^{-3}$	$6.26 \cdot 10^{-5}$
	Dist to Mean of Speed of Tilt-x Change Freq.	12.56	**$6.36 \cdot 10^{-28}$**	$2.27 \cdot 10^{-4}$	$4.35 \cdot 10^{-5}$	$4.34 \cdot 10^{-4}$	$2.04 \cdot 10^{-4}$
	Bandwidth of Speed of Tilt-y Change Freq.	6.19	**$2.64 \cdot 10^{-9}$**	$3.45 \cdot 10^{-3}$	$2.72 \cdot 10^{-4}$	$3.72 \cdot 10^{-3}$	$3.36 \cdot 10^{-4}$
	Median of Power Spectral of Speed of Tilt-y Change Freq.	-9.66	**$8.93 \cdot 10^{-19}$**	$3.35. \cdot 10^{-3}$	$2.30 \cdot 10^{-5}$	$3.30. \cdot 10^{-3}$	$4.38 \cdot 10^{-5}$
	Dist to Mean of Speed of Tilt-y Change Freq.	7.97	**$7.24 \cdot 10^{-14}$**	$2.26 \cdot 10^{-4}$	$2.79 \cdot 10^{-5}$	$2.85 \cdot 10^{-4}$	$8.57 \cdot 10^{-5}$

Supplementary Table 5: A comparison between the two groups of writers (*D dataset* and *TD dataset*) for every feature based on the tilt between the pen and tablet surface (see Supplementary Figure 5). For each feature, we report the mean and standard deviation of the two groups as well as the t-statistic and p-value.

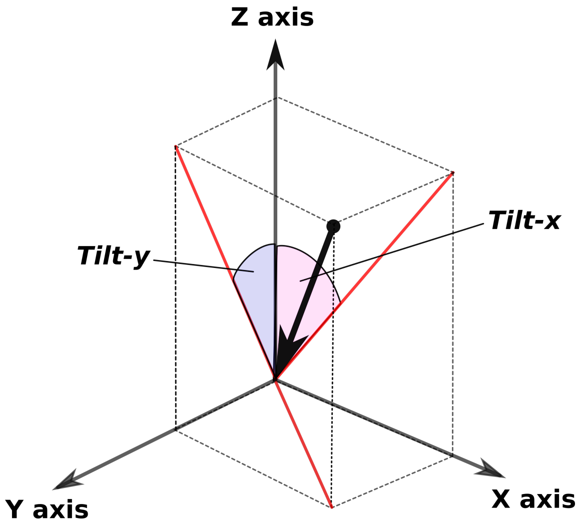

Supplementary Figure 5: The two angles (tilt-x and tilt-y) recorded for the pen. The black arrow represents the pen, and the red segments represent its projection on the XZ and YZ planes, respectively.

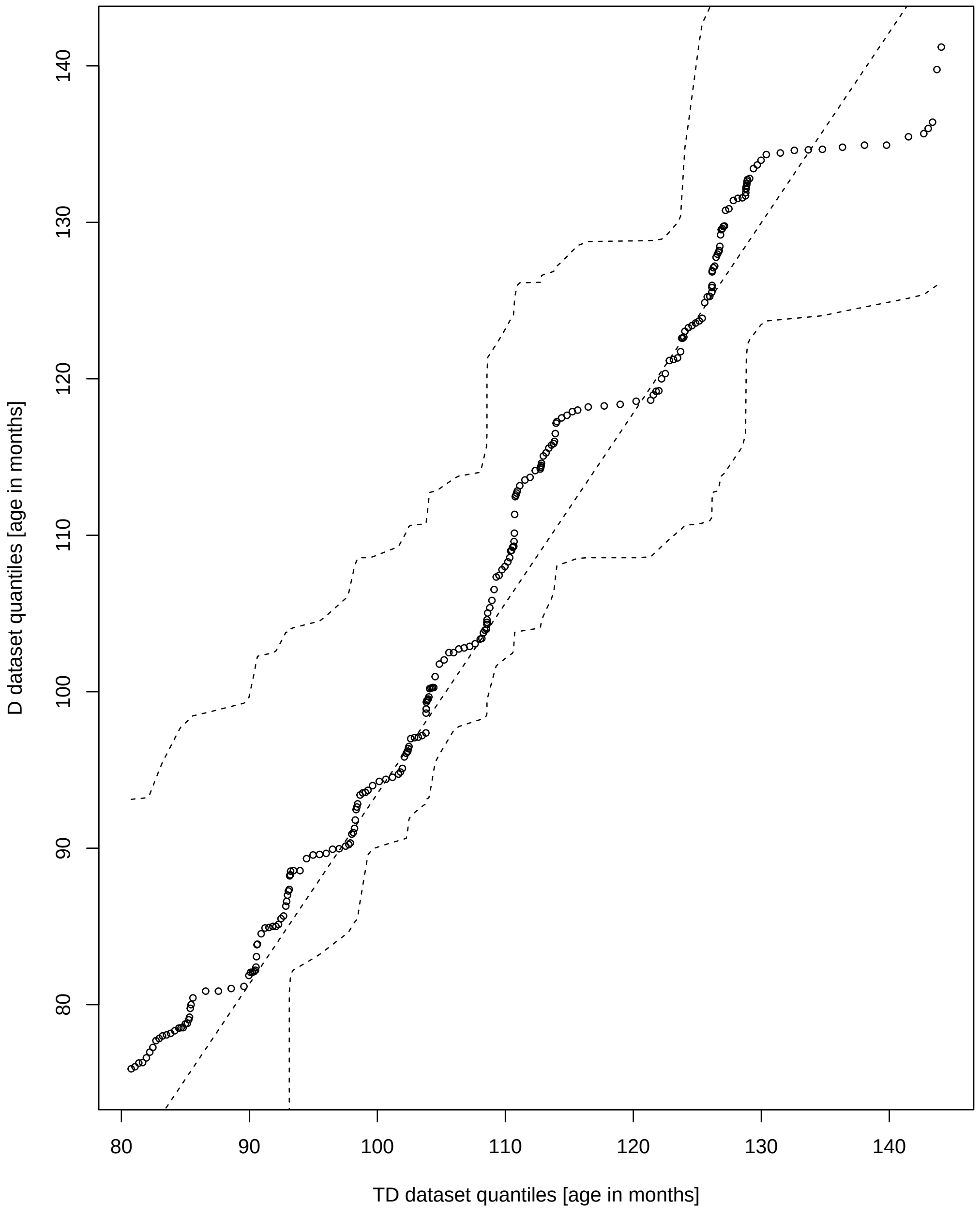

Supplementary Figure 6: Quantiles of the *TD dataset* (x-axis) against the quantiles of the *D dataset* (y-axis). The points are closed from the diagonal dashed line and included in the confidence bounds, showing the similarity of the two distributions.

	BHK Item	Corresponding Feature
**Letter**	Variation in Height of Small Letters (a, c, e, i, m, n, o, r, s, u ,v, w, x)	✗
	Incorrect Relative Height of letters	✗
	Distorted Letters	✗
	Ambiguous Shapes of Letters	✗
**Text**	Handwriting Size	Handwriting Size
	Margin Shifted to the Right	✗
	Non Linear Lines	Moment of Handwriting
	Words too Narrowed	Space Between Words
	Chaotic Writing (trace not fluid, too many change of directions during handwriting)	Median of Power spectral of Tremor Frequencies*, Bandwidth of Tremor Frequencies*, Median of Power Spectral of Speed Frequencies*, ...
	Loss of Links between Letters	In Air Time Ration*, Std. of Speed*, Std. of pressure*, ...
	Superposed Letters	Handwriting Density*
	Modified Letters	Handwriting Density*
	Shaky Handwriting	Bandwidth of Tremor Frequencies, Median of Power spectral of Tremor Frequencies

Supplementary Table 6: Mapping between the extracted features and the BHK items. *represents features which are not mapped directly to the BHK item but which are likely to explain a similar concept. The category of the BHK features is written in the left column.
